# Real-World Assessment of Weight Change in African American Females and Hispanics with HIV-1 After Initiating Integrase Strand-Transfer Inhibitors or Protease Inhibitors

**DOI:** 10.36469/001c.30184

**Published:** 2022-01-03

**Authors:** Yen-Wen Chen, David Anderson, Christopher D. Pericone, Prina Donga

**Affiliations:** 1 Janssen Scientific Affairs, LLC, Titusville, NJ, USA

**Keywords:** weight gain, HIV, integrase inhibitors, antiretroviral therapy, women

## Abstract

**Background:** Studies have shown an increase in weight among people living with HIV (PLWH) who initiated integrase strand transfer inhibitors (INSTI). However, weight gain with INSTI-based regimens vs other regimens in females or racial/ethnic minorities is poorly understood.

**Objective:** This study assessed differences in weight gain among treatment-naïve, female, African Americans and Hispanics after initiating INSTI-based vs protease inhibitor (PI)-based regimens.

**Methods:** This retrospective, observational cohort study included data from the Optum® deidentified Electronic Health Record Database. Female African Americans or Hispanics initiating INSTI- or PI-based regimens between January 1, 2015, and December 31, 2018 (first prescription was index date), with ≥12-month baseline and follow-up periods, ≥1 weight measure during each period, and no prior antiretroviral (ARV) use were included. Inverse probability of treatment weighting was used to reduce selection bias and improve cohort comparability. Multivariable models were used to compare absolute weight/body mass index (BMI) changes and proportion of patients with weight/BMI increases from pre- to post-index (last measure between the 4th and 12th months post-index).

**Results:** Weighted cohorts included 3407 African American females (INSTI, 1704; PI, 1703) and 3711 Hispanics (INSTI, 1865; PI, 1846) PLWH. Mean time to follow-up weight measure was ~9.5 months. Among female African Americans, INSTI initiators had a 1.5 kg greater mean weight gain (2.1 kg vs 0.6 kg; P = 0.033), and a higher proportion with ≥5% weight gain (32% vs 29%; odds ratio [OR]=1.2; 95% CI [1.0-1.4]) than PI initiators. Among Hispanics, INSTI and PI initiators had similar mean increases in weight (2.1 and 1.8 kg, respectively), but INSTI initiators had a higher proportion with ≥5% weight gain (31% vs 27%; OR=1.2; 95% CI [1.1-1.4]). Female African American INSTI initiators were more likely to shift from normal or overweight to a worse BMI classification. Hispanic INSTI initiators were less likely to shift from normal BMI to overweight but more likely to shift from normal or overweight to obese.

**Conclusion:** In a real-world setting, INSTI-based regimens were associated with greater weight gain for treatment-naïve female African Americans, compared with PI-based regimens. Differences between regimens were less consistent for Hispanics. These results may inform ARV choice for PLWH who are at risk for ARV-related weight gain.

## INTRODUCTION

At the end of 2019, the Centers for Disease Control and Prevention reported 36 000 new diagnoses of HIV and >1 million people living with HIV (PLWH) in the United States.^1^ Moreover, African American and Hispanics are disproportionately affected by HIV, accounting for 40% and 25% of PLWH in the United States, respectively,^1^ and having generally poorer outcomes than white PLWH, including lower rates of being on antiretroviral (ARV) treatment or achieving viral load suppression.^2^ As another consideration in HIV care, as the life expectancy of PLWH has increased due to ARV therapies,^3^ the proportion of PLWH ≥50 years old has increased from 46% in 2015 to 52% in 2019.^1^ As the mean age of PLWH increases, this population is at a greater risk of developing chronic conditions such as obesity, hypertension, cardiovascular disease, and diabetes, which are especially prevalent among PLWH.^4–8^ The risk of chronic diseases is of special concern for African Americans and Hispanics, who have a higher prevalence of diabetes and cardiovascular disease and associated risk factors, such as obesity and hypertension.^9–14^ Aging and HIV status compound these risks.

Initiation of ARV therapy has been reported to lead to weight gain within approximately the first year, with several studies reporting mean increases of a few kilograms and 20%-40% of patients shifting to a higher category of body mass index (BMI) (eg, normal to overweight, overweight to obese).^15–17^ Among ARV therapies, integrase strand transfer inhibitor (INSTI)–based regimens have been associated with greater weight gain than regimens based on protease inhibitors (PIs) or non-nucleoside reverse transcriptase inhibitor in real-world studies of treatment naïve patients^18–20^ and patients who switch their ARV therapy to an INSTI-based regimen.^21,22^ Within the INSTI class, dolutegravir and bictegravir have been associated with greater weight gain than other INSTIs.^17,18,20^ Tenofovir alafenamide (TAF), a nucleoside reverse transcriptase inhibitor (NRTI) commonly included in ARV regimens, has also been associated with ARV-associated weight gain, especially in comparison to tenofovir disoproxil fumarate.^20,23^ However, the impact of TAF, particularly in relation to INSTI-based regimens, remains poorly understood.^24^ Nevertheless, data from recent clinical trials have demonstrated that even among regimens without TAF, INSTI-based regimens are associated with the greatest weight gain.^20,23,25^ Importantly, ARV-related weight gain has been associated with increased risk for diabetes and cardiovascular disease among PLWH (myocardial infarction [MI], stroke, coronary conditions).^5,8^

Recent US Department of Health and Human Services guidelines highlight concerns related to weight gain associated with certain ARV therapies, such as INSTI-based regimens, and encourage providers to consider the tolerability profile of these agents when selecting an appropriate regimen and to monitor for weight gain.^26^ Current guidelines also specify that weight gain may be especially prevalent in certain patient populations (eg, women, African Americans, and Hispanics), consistent with recent reports of gender and racial differences in the risk of weight gain upon initiating INSTI-based regimens.^20,22,27,28^ Recent studies have shown that African Americans and women are at a higher risk of weight gain with INSTI-based regimens vs other regimens,^22,29^ but data specifically focused on African American females and Hispanics are lacking. Therefore, the current study aimed to understand the difference in weight gain among treatment-naïve female African American PLWH or Hispanic PLWH after initiating INSTI-based vs PI-based ARV regimens.

## METHODS

### Data Source

Patient-level records from the Optum® Pan-Therapeutic Deidentified Electronic Health Records (EHR) database were obtained. This database contains deidentified longitudinal data on diagnostic procedures, medications, laboratory results, outpatient visits, hospitalizations, clinical notes, and patient outcomes, primarily from integrated delivery networks for 80 million US patients (≥7 million patients from each census region).

### Study Period and Population

**Figure 1.**
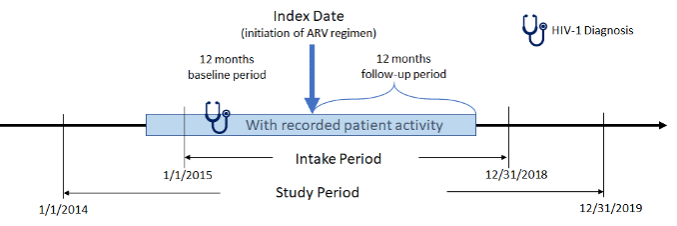
Study Design and Time Frame

The study period for this retrospective, observational, matched-cohort study was January 1, 2014, to December 31, 2019 (Figure 1). Female African American and male and female Hispanic PLWH with ≥1 written prescription for INSTI- or PI-based ARV regimens between January 1, 2015, and December 31, 2018, (intake period) were included. Index date was defined as the date of the earliest written ARV prescription during the intake period. Additional study inclusion criteria were: age ≥18 years at index, ≥365 days of EHR activity pre-/post-index, ≥1 diagnosis for HIV-1 during the year prior to index date (baseline period), and ≥1 baseline (between 12 months pre- and 30 days post-index) and follow-up (between 4th and 12th month post-index) measure for either weight or BMI. Exclusion criteria were: ≥1 HIV-2 diagnosis or any evidence of pregnancy during the study; ≥1 prescription for any ARV during the baseline period; ≥1 diagnosis for liver disease or chronic kidney disease (stage 4 and above) or ≥1 laboratory report for creatinine clearance <30 min/ml during the 12-month baseline period; and inconsistent/missing data on gender or birth year. Although it would be useful to analyze female Hispanics alone, sample size considerations did not allow this approach.

### Identification of Index Regimen

Treatment-naïve patients with HIV-1 are generally treated with an ARV regimen comprising two nucleoside reverse transcriptase inhibitors (NRTIs) in combination with a third ARV agent from other ARV drug classes (INSTI, PI, or non-NRTI. Current ARV regimens are formulated as fixed-dose combinations (FDC) which contain a combination of two or more ARV agents in a single tablet, and multitablet regimen formulations. In the current study, INSTI- or PI-based regimens identified on the index date were defined as the index regimen. Patients were classified as INSTI initiators if the index ARV regimen included dolutegravir, raltegravir, bictegravir, or elvitegravir. Patients were classified as PI initiators if the index ARV regimen included atazanavir or darunavir. Patients not taking an FDC INSTI or PI were required to have prescriptions for ≥2 distinct NRTIs or ≥1 NRTI FDC within 14 days of the index date. Patients with >1 INSTI or PI drug identified on the index date were excluded.

### Outcomes

An intent-to-treat approach was used to evaluate change in weight and BMI within 12 months post initiation of INSTI- vs PI-based regimens in treatment-naïve (1) female African Americans and (2) male and female Hispanics. The primary outcomes were absolute change and proportion of patients with ≥5% change in weight and BMI from baseline to follow-up. Secondary outcomes included proportion of patients with weight change ≥10 kg, ≥10%, ≥20%, and proportion of patients who shifted to a different BMI category. All outcomes were also evaluated by index BMI value category (<25 kg/m^2^ vs. ≥25 kg/m^2^).

### Variables

Variables included demographic characteristics (age, reported gender [for Hispanic cohort only], race [for Hispanic cohort only], region, insurance type, and index year), clinical characteristics (Quan-Charlson Comorbidity Index [QCI], comorbidities [see diagnostic codes in **Supplemental Code Lists**], and medication use [see medication codes in **Supplemental Code Lists**]).

### Data Analysis

Univariate statistics were used to descriptively analyze demographic characteristics, clinical characteristics, and outcomes of interest. Frequencies and percentages were reported for categorical variables; means and SD were reported for continuous variables.

**Inverse probability of treatment weighting approach:** The inverse probability of treatment weighting approach (IPTW) was used to control for selection bias and improve cohort comparability. To account for the effect on weight change from both the type of index regimen and the presence of TAF, IPTW was conducted in four treatment cohorts: PI with TAF, PI without TAF, INSTI with TAF and INSTI without TAF. A logistic regression model was used to calculate the propensity scores for each patient in the four treatment cohorts, with PI serving as the reference group for the generation of propensity scores. Independent variables in the model for female African Americans included age, baseline weight, baseline QCI, number of cardiovascular disease risk factors (prediabetes/glucose intolerance, type 2 diabetes, MI, congestive heart failure, hypertension and hyperlipidemia) at baseline and number of potentially weight modifying drugs (diabetes therapies, psychiatric/neurologic therapies, hormone therapy/contraception, stimulants/appetite suppressants, and anti-hypertensives) used at baseline. Independent variables in the model for Hispanics included age, gender, baseline weight, baseline QCI, and number of potentially weight-modifying drugs used at baseline. Sample size considerations did not allow the inclusion of the number of cardiovascular risk factors as an independent variable for Hispanics, but inclusion of number of potentially weight-modifying drugs (especially diabetes therapies and antihypertensives) were used to serve as a proxy for cardiovascular risk. The inverse probability of treatment weight was calculated as the inverse of the propensity score. After the four treatment cohorts were balanced (standard difference <10%), PI with TAF and PI without TAF cohorts were grouped into one PI cohort. The same grouping was done for the INSTI cohort. The comparisons of outcomes were done between PI vs INSTI cohorts.

**Outcome models:** Multivariable regression models were used to compare the outcomes of interest between IPTW treatment cohorts. Ordinary least-squares models were used to model absolute and expected percent change in weight and BMI, as a function of the independent variable (INSTI- vs PI-based regimens) and covariates. *P* values and 95% confidence intervals (CI) were calculated. The expected proportion of patients having weight/BMI increases as a function of the independent variable (INSTI- vs. PI-regimens) and covariates was modeled using logistic regression. A *P* value <0.05 was considered statistically significant for all analyses.

**Analytical software:** All analyses used SAS Enterprise Guide, version 7 (SAS Institute, Cary, NC).

## RESULTS

### Baseline Demographics and Characteristics of African American Females

Of the 7271 patients with ≥1 prescription for a INSTI-based or PI-based regimen during the intake period and ≥1 HIV-1 diagnosis within the prior 12 months, 852 were female African Americans (INSTI, 688; PI, 164). Baseline demographics and clinical characteristics between PI and INSTI cohorts were similar **(Supplemental Table S1)**. IPTW was used to create weighted cohorts of similar size (INSTI, 1704; PI, 1703) that were balanced with regards to key baseline demographic and clinical variables (Table 1). Among the weighted INSTI and PI cohorts, mean age was 46.9 and 47.2 years, mean weight at baseline was 82.8 and 83.2 kg, and mean BMI at baseline was 30.8 and 30.6 kg/m^2^, respectively. Mean time to follow-up weight measure was 295 days and 284 days (*P*=0.059) for the INSTI and PI cohorts, respectively. The INSTI and PI cohorts had the same mean QCI score (3.2) and mean number of cardiovascular conditions (0.8). Similar proportions had a baseline diagnosis of AIDS (INSTI, 66.2%; PI, 63.0%;​), hypertension (INSTI, 36.4%; PI, 30.6%), type 2 diabetes (INSTI, 15.4%; PI, 16.2%) or hyperlipidemia (INSTI, 14.3%; PI, 14.5%). Among those with insurance plan information, the most common types were Medicaid (INSTI, 13.4%; PI, 21.8%) and Commercial (INSTI, 17.6%; PI, 9.8%).

**Table 1.** Baseline Demographic and Clinical Characteristics for Female African American and Hispanic Patients

	**Female African American**			**Hispanic**		
	**PI (weighted n=1703)**	**INSTI (weighted n=1704)**	**|d|^a^**	**PI (weighted n=1846)**	**INSTI (weighted n=1865)**	**|d|^a^**
**Age, mean (SD)**	47.2 (10.3)	46.9 (11.3)	2.6%	43.3 (10.9)	43.0 (13.3)	2.4%
**Female, n (%)**	N/A	N/A	N/A	475 (25.7)	462 (24.8)	2.2%
**Race, n (%)**						
African American	N/A	N/A	N/A	51 (2.8)	101 (5.4)	13.6%
Asian	N/A	N/A	N/A	0 (0.0)	6 (0.4)	8.3%
Caucasian	N/A	N/A	N/A	1126 (61.0)	959 (51.4)	19.5%
Other/unknown	N/A	N/A	N/A	669 (36.2)	799 (42.8)	13.5%
**Insurance plan type, n (%)**						
Commercial	167 (9.8)	299 (17.6)	22.8%	291 (15.8)	469 (25.2)	23.5%
Medicaid	371 (21.8)	229 (13.4)	22.0%	81 (4.4)	169 (9.0)	18.6%
Medicare	164 (9.7)	69 (4.0)	22.4%	68 (3.7)	85 (4.6)	4.5%
Other**^b^**	144 (8.5)	176 (10.4)	6.5%	439 (23.8)	360 (19.3)	18.1%
Missing	858 (50.3)	930 (54.6)	8.6%	967 (52.4)	783 (42.0)	20.9%
**Baseline weight, mean (SD)**	83.2 (22.6)	82.8 (25.4)	2.0%	77.2 (20.2)	78.5 (18.1)	7.1%
**Baseline BMI, mean (SD)**	30.6 (8.3)	30.8 (9.2)	2.0%	27.2 (6.1)	27.4 (6.1)	2.7%
**Baseline BMI category, n (%)**						
Underweight (BMI<18.5)	49 (2.9)	62 (3.7)	4.3%	107 (5.8)	54 (2.9)	14.4%
Normal (BMI 18.5-24.9)	319 (18.7)	414 (24.3)	13.6%	526 (28.5)	634 (34.0)	11.9%
Overweight (BMI 25.0-29.9)	464 (27.3)	389 (22.8)	10.2%	681 (36.9)	608 (32.6)	8.9%
Obese (BMI ≥30)	806 (47.3)	776 (45.6)	3.5%	437 (23.7)	502 (26.9)	7.4%
Missing	65 (3.8)	63 (3.7)	0.6%	95 (5.1)	68 (3.6)	7.3%
**Baseline comorbidities**						
QCI score, mean (SD)	3.2 (2.5)	3.2 (2.3)	1.0%	3.1 (2.1)	3.1 (2.2)	1.2%
**No. of CVD risk conditions, mean (SD)**	0.8 (1.0)	0.8 (1.0)	0.6%	0.6 (0.9)	0.5 (0.9)	3.9%
**Individual conditions, n (%)**						
T2DM	277 (16.2)	263 (15.4)	2.2%	239 (12.9)	232 (12.5)	1.5%
Hypertension	522 (30.6)	620 (36.4)	12.2%	317 (17.2)	310 (16.6)	1.4%
Hyperlipidemia	247 (14.5)	244 (14.3)	0.5%	337 (18.3)	307 (16.5)	4.7%
AIDS	1,074 (63.0)	1,127 (66.2)	6.5%	1,222 (66.2)	1,283 (68.8)	5.6%
**No. of drugs used at baseline, mean (SD)^c^**	0.6 (1.0)	0.7 (1.2)	2.5%	0.4 (0.7)	0.4 (0.9)	2.8%

Abbreviations: BMI, body mass index; CVD, cardiovascular diseases; INSTI, integrase inhibitors; PI, protease inhibitors; QCI, Quan Charlson Comorbidity Index; T2DM, type 2 diabetes mellitus.

^a^ Standard difference, with |d| ≥10% being considered as significant.

^b^ Includes Multiple, Uninsured, Unknown, or Other.

Among the INSTI cohort, 62 (3.6%) were underweight at baseline, 414 (24.3%) had normal bodyweight, 389 (22.8%) were overweight, and 776 (45.5%) were obese (BMI ≥30 kg/m^2^). Among the PI cohort, 49 (2.9%) patients were underweight at baseline (BMI <18.5 kg/m^2^), 319 (18.7%) had normal bodyweight (BMI 18.5-24.9 kg/m^2^), 464 (27.2%) were overweight (BMI 25.0-29.9 kg/m^2^), and 806 (47.3%) were obese (Table 2).

**Table 2.** Index BMI Category and Proportion of African American Female Patients With BMI Category Shifts During Follow-up

**Index BMI Category**	PI-Based Regimens: Post-index BMI Category, n (%)*			
	**Underweight** **,** **n=23**	**Normal,** **n=360**	**Overweight,** **n=556**	**Obese,** **n=699**
Underweight (BMI <18.5), n=49	8 (16.3)	38 (77.6)	0 (0.0)	3 (6.1)
Normal (BMI 18.5-24.9), n=319	15 (4.7)	237 (74.3)	60 (18.8)	7 (2.2)
Overweight (BMI 25.0-29.9), n=464	0 (0.0)	85 (18.3)	359 (77.4)	20 (4.3)
Obese (BMI ≥30), n=806	0 (0.0)	0 (0.0)	137 (17.0)	669 (83.0)
				
**Index BMI Category**	INSTI-Based Regimens: Post-index BMI Category, n (%)*			
	**Underweight,** **n=43**	**Normal,** **n=359**	**Overweight,** **n=440**	**Obese,** **n=798**
Underweight (BMI <18.5), n=62	19 (30.6)	41 (66.1)	2 (3.2)	0 (0.0)
Normal (BMI 18.5-24.9), n=414	24 (5.8)	265 (64.1)	96 (23.3)	28 (6.8)
Overweight (BMI 25.0-29.9), n=389	0 (0.0)	47 (12.1)	275 (70.7)	67 (17.2)
Obese (BMI ≥30), n=776	0 (0.0)	6 (0.8)	67 (8.6)	703 (90.6)

Abbreviations: BMI, body mass index; INSTI, integrase strand transfer inhibitor; PI, protease inhibitor.

^a^ Proportions reflect total for each row.

### Weight and BMI Changes in African American Females

**Figure 2.**
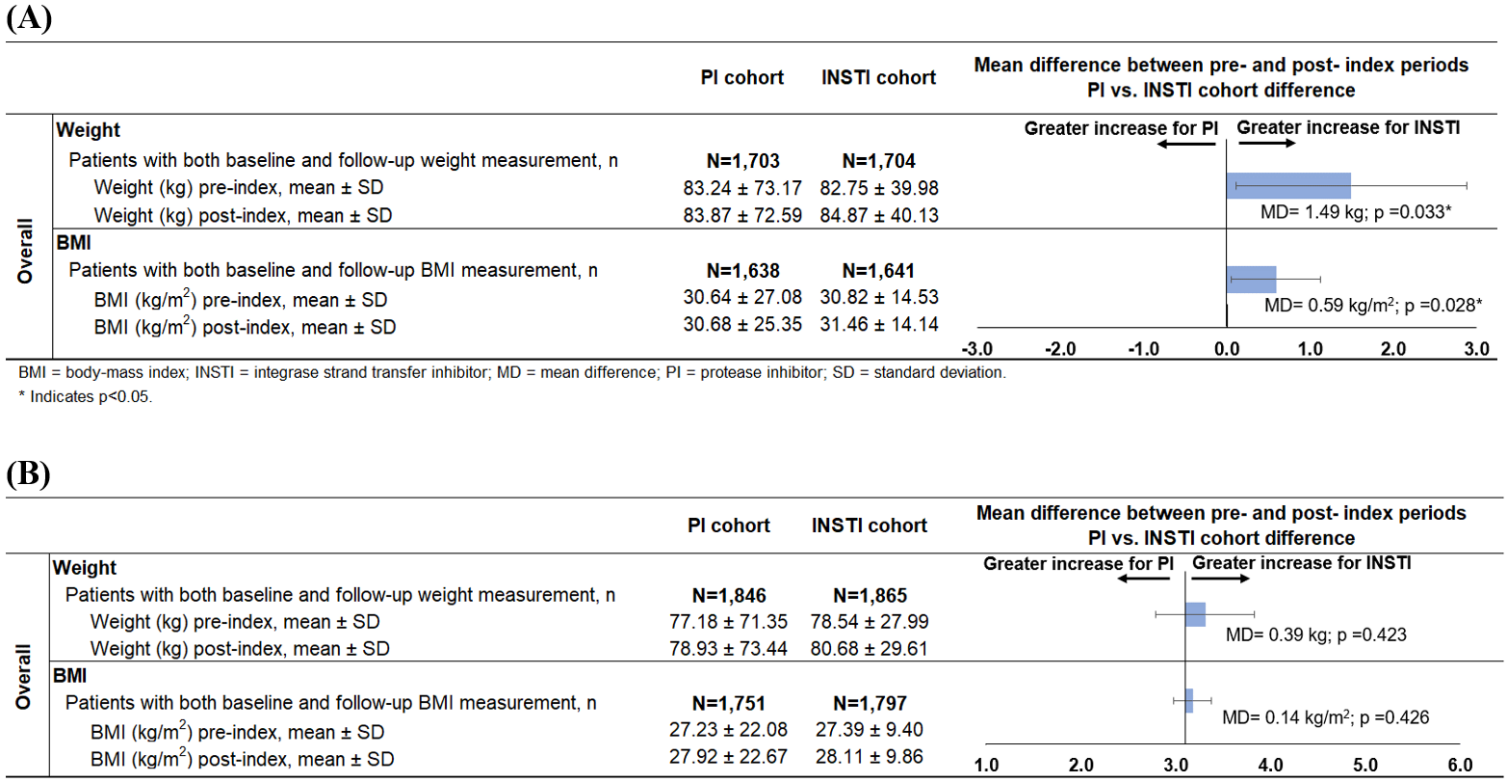
Mean Change in Weight in Female African Americans **(A)** or Hispanics **(B)**

**Figure 3.**
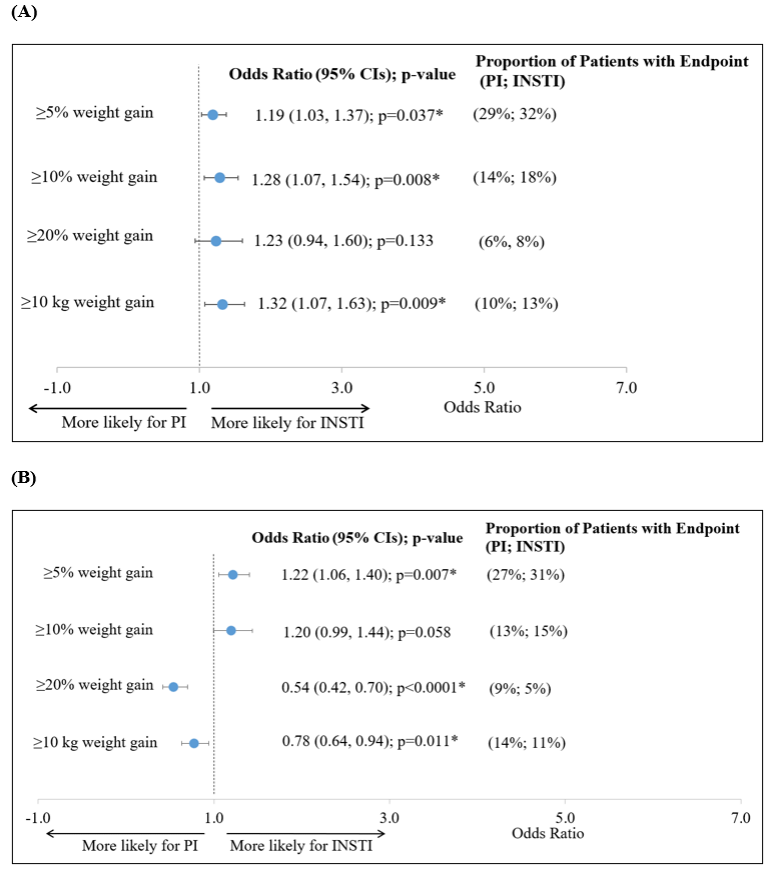
Odds Ratio of Weight Gain in Female African Amerians **(A)** or Hispanics **(B)** Abbreviations: CIs, confidence intervals; INSTI, integrase strand transfer inhibitor; PI, protease inhibitor. * *P*&lt;0.05.

After a mean follow-up of ~ 9.5 months, African American females initiating INSTI-based regimens had a 1.5 kg greater mean weight gain (2.1 kg vs 0.6 kg; *P*=0.033) and a 0.6 kg/m^2^ greater mean BMI increase (0.6 kg/m^2^ vs 0.0 kg/m^2^; *P*=0.028), compared with PI initiators (Figure 2A). When stratified by the presence of TAF, the mean±SD weight gain was 2.6±17.7 kg vs 1.6±12.2 kg for those initiating INSTI-based regimens with or without TAF, respectively. The mean ± SD weight gain was 1.5±54.1 kg vs –0.3±29.8 kg for those initiating PI-based regimens with or without TAF, respectively (data not shown). Similarly, a greater proportion of INSTI initiators experienced ≥5% weight gain (32% vs 29%; OR=1.2; 95% CI [1.0–1.4]), ≥10% weight gain (18% vs 14%; OR=1.3; 95% CI [1.1-1.5]), or ≥10 kg weight gain (13% vs 10%; OR=1.3; 95% CI [1.1-1.6]) than PI initiators (Figure 3A).

### BMI Category Shifts in African American Females

Among female African American INSTI initiators with normal baseline weight, 23.3% became overweight (BMI ≥25 kg/m^2^) and 6.8% became obese (BMI ≥30 kg/m^2^) during follow-up. Among normal-weight PI initiators, 18.8% and 2.2% became overweight or obese, respectively, during follow-up. Among overweight INSTI initiators, 17.2% became obese during follow-up, as compared with 4.3% of PI initiators (Table 2).

### Weight and BMI Changes in African American Females by Baseline BMI

Among female African Americans with baseline BMI <25 kg/m^2^ or ≥25 kg/m^2^, there were no significant differences between INSTI and PI initiators regarding the primary endpoints, ie, mean change in weight or BMI **(Supplemental Figure S1)** or proportion experiencing 5% increase in weight **(Supplemental Figure S2).**

### Baseline Demographic and Clinical Characteristics for Hispanics

Of the 7271 patients with ≥1 prescription for an INSTI or PI-based regimen during the intake period and ≥1 HIV-1 diagnosis within the prior 12 months, 932 patients were Hispanic (INSTI, 783; PI, 149). Baseline demographics and clinical characteristics between INSTI and PI cohorts were similar **(Supplemental Table S2).** IPTW was used to create weighted cohorts of similar size (INSTI, 1865; PI, 1846) that were balanced with regard to key baseline demographic and clinical variables Table 1. Among the weighted INSTI and PI cohorts, mean age was 43.0 and 43.3 years, and 24.8% and 25.7% were females, respectively. Male and female Hispanics were not analyzed separately due to sample size considerations. Mean weight at baseline was 78.5 and 77.2 kg, and mean BMI at baseline was 27.4 and 27.2 kg/m^2^, respectively. Mean time to follow-up weight measure was 284 days and 282 days (*P*=0.733) for the INSTI and PI cohorts, respectively. The INSTI and PI cohorts had the same mean QCI score (3.1) and similar number of mean cardiovascular conditions (0.5 vs 0.6). Similar proportions had a baseline diagnosis of AIDS (INSTI, 68.8%; PI, 66.2%), hypertension (INSTI, 16.6%; PI, 17.2%), type 2 diabetes (INSTI, 12.5% ; PI, 12.9%) or hyperlipidemia (INSTI, 16.5%; PI, 18.3%). Information about plan type was missing for 42.0% and 52.4% of INSTI and PI-based cohorts, respectively. Among those with insurance plan information, the most common types were Commercial (INSTI, 25.2%; PI, 15.8%) and Other, which included Multiple, Uninsured, Unknown, or Other (INSTI, 19.3%; PI, 23.8%).

Among the INSTI cohort, 54 (2.9%) were underweight at baseline, 634 (34.0%) had normal bodyweight, 608 (32.6%) were overweight, and 502 (26.9%) were obese (BMI ≥30 kg/m^2^). Among the PI cohort, 107 (5.8%) were underweight at baseline (BMI: <18.5 kg/m^2^), 526 (28.5%) had normal bodyweight (BMI: 18.5–24.9 kg/m^2^), 681 (36.9%) were overweight (BMI: 25.0–29.9 kg/m^2^), and 437 (23.7%) were obese (Table 3).

**Table 3.** Index BMI Category and Proportion of Hispanic Patients With BMI Category Shifts During Follow-up

**Index BMI Category**	**PI-Based Regimens: Post-index BMI Category, n(%)^a^**			
	**Underweight,** **n=109**	**Normal,** **n=533**	**Overweight,** **n=643**	**Obese,** **n=466**
Underweight (BMI <18.5), n=107	78 (72.7)	29 (27.3)	0 (0.0)	0 (0.0)
Normal (BMI 18.5-24.9), n=526	12 (2.3)	344 (65.4)	166 (31.5)	4 (0.8)
Overweight (BMI 25.0-29.9), n=681	19 (2.8)	156 (22.9)	439 (64.5)	67 (9.8)
Obese (BMI ≥30), n=437	0 (0.0)	4 (0.8)	38 (8.7)	395 (90.5)

Index BMI Category	INSTI-Based Regimens, Post-index BMI Category, n (%)^a^			
	Underweight, n=31	Normal, n=588	Overweight, n=607	Obese, n=570
Underweight (BMI <18.5), n=54	21 (38.6)	20 (38.2)	12 (23.2)	0 (0.0)
Normal (BMI 18.5-24.9), n=634	8 (1.2)	482 (76.1)	132 (20.9)	12 (1.8)
Overweight (BMI 25.0-29.9), n=608	2 (0.3)	77 (12.6)	411 (67.6)	118 (19.4)
Obese (BMI ≥30), n=502	0 (0.0)	9 (1.9)	52 (10.4)	440 (87.7)

Abbreviations: BMI, body mass index; INSTI , integrase strand transfer inhibitor; PI, protease inhibitor.

^a^ Proportions reflect total for each row.

### Weight and BMI Changes in Hispanics

After a mean follow-up of ~9.5 months, Hispanic patients initiating INSTI or PI-based regimens had similar mean increases in weight (2.1 and 1.8 kg, respectively) and BMI (0.7 and 0.6 kg/m^2^, respectively) (Figure 2B). When stratified by the presence of TAF, the mean ± SD weight gain was 2.5±7.9 kg and 1.8±7.1 kg for those initiating INSTI-based regimens with or without TAF, respectively. The mean ± SD weight gain was 2.9±6.8 kg and 0.6±7.3 kg for those initiating PI-based regimens with or without TAF, respectively (data not shown). INSTI initiators were more likely than PI initiators to experience ≥5% weight gain (31% vs 27%; OR=1.2; 95% CI [1.1-1.4]) (Figure 3B) or ≥5% BMI increase (30% vs 26%; OR=1.2; 95% CI [1.0-1.4]) (data not shown), but less likely to experience weight gains ≥20% (5% vs 9%; OR=0.5; 95% CI [0.4-0.7]) or ≥10 kg (11% vs 14%; OR=0.8; 95% CI [0.6-0.9]) (Figure 3B).

### BMI Category Shifts in Hispanics

Among Hispanic INSTI initiators with normal baseline weight, 20.9% became overweight (BMI ≥25 kg/m^2^) and 1.8% became obese (BMI ≥30 kg/m^2^) during follow-up. Among normal weight PI initiators, 31.5% and 0.8% became overweight or obese, respectively, during follow-up. Among overweight INSTI initiators, 19.4% became obese during follow-up, as compared with 9.8% of PI initiators (Table 3).

### Weight and BMI Changes in Hispanics by Baseline BMI

Among Hispanic patients with baseline BMI <25 kg/m^2^, PI initiators had a 1.7 kg greater mean weight gain (4.2 kg vs 2.5 kg; *P*=0.035), but a similar mean BMI increase (1.4 kg/m^2^ vs 1.0 kg/m^2^; *P*=0.158), compared with INSTI initiators **(Supplemental Figure S3A).** In contrast, Hispanic patients with baseline BMI ≥25 kg/m^2^ who initiated INSTI-based regimens had 1.6 kg greater mean weight gain (2.0 kg vs 0.3 kg; *P*=0.005) and 0.5 kg/m^2^ greater mean BMI increase (0.5 kg/m^2^ vs 0.1 kg/m^2^; *P*=0.043), compared with PI initiators **(Supplemental Figure S3B).**

Among Hispanic patients with baseline BMI <25 kg/m^2^, INSTI initiators were less likely than PI initiators to experience ≥5% weight gain (34% vs 40%; OR=0.8; 95% CI [0.6-1.0]) **(Supplemental Figure S4A)** or ≥5% increase in BMI (34% vs 39%; OR=0.8; 95% CI [0.6-1.0]) (data not shown). In contrast, among patients with baseline BMI ≥25 kg/m^2^, a greater proportion of INSTI initiators experienced ≥5% weight gain (30% vs 21%; OR=1.6; 95% CI [1.3-2.0]) **(Supplemental Figure S4B)** or ≥5% increase in BMI (30% vs. 21%; OR=1.6; 95% CI [1.3-1.9] (data not shown).

## DISCUSSION

After a mean ~9.5-month follow-up, female African American INSTI initiators had a 1.5 kg greater mean weight gain, 0.6 kg/m2 greater mean BMI increase, and greater odds of ≥5% weight gain or ≥5% BMI increase vs PI initiators. Similar trends were observed in female African Americans for weight gains ≥10 kg or ≥10%; however, differences were not significant for >20% weight gain, possibly due to the small number of participants with extreme gains. In the Hispanic study population, mean weight gain was not significantly different between regimens (INSTI, 2.1 kg; PI, 1.8 kg), although the risk of ≥5% weight gain was greater for INSTI initiators vs PI initiators, while the risk of ≥20% or ≥10 kg weight gain was lower in INSTI initiators than PI initiators. INSTI initiators with baseline BMI ≥25 kg/m^2^ had greater absolute mean weight gain and BMI increase than PI initiators for both study populations, whereas for INSTI initiators with baseline BMI <25 kg/m^2^, absolute mean weight gain and BMI increases were higher for female African Americans and lower for Hispanics. The majority of subjects in our study were overweight (female African Americans, 70%; Hispanics, 60%), underscoring the potential importance of our findings. Moreover, hypertension (female African Americans, 33%; Hispanics 17%) and type 2 diabetes (female African Americans, 16%; Hispanics, 13%), which are important risk factors for complications such as MI, stroke, and chronic renal disease, were common in our study population, consistent with reports of their high prevalence in PLWH.^6,7^

These findings are consistent with previous clinical and real-world studies reporting increased weight or BMI within approximately 1 year of initiating ARV, and greater weight gain with INSTIs vs other regimens, especially among females and African Americans.^20,22,27–29^ In a recent real-world study of treatment naïve PLWH, Chen et al reported a higher risk of 5% weight gain for INSTI vs. PI initiators among the overall study population (1.3 kg greater mean weight gain for INSTI) and among females (2.5 kg greater weight gain for INSTI).^29^ A similar real-world study using a different database reported a higher risk of 5% weight gain for INSTI vs. PI initiators overall (1.5 kg greater weight gain for INSTI), but the differences did not reach statistical significance among females, perhaps due to smaller sample size.^30^ In a pooled analysis of 8 clinical trials, Sax et al reported approximately 1.5 kg greater mean weight gain for INSTIs vs. PIs, with female and African Americans/Black patients having greater risk of ≥10% weight gain.^20^ Similarly, a recent prospective, observational study by Kline et al of military personnel with HIV reported that overweight African American men initiating INSTI-based regimens had greater mean BMI increase at 2 years than overweight White men (1.85 kg/m^2^/yr, P = 0.007).^31^ Lake et al recently reported that among virally suppressed PLWH who switched to INSTI regimens, Black race and female sex were associated with greater weight gain.^28^

However, the literature reports inconsistent results regarding baseline BMI as a risk factor for ARV-related or INSTI-related weight gain. Kline et al found no significant difference in weight gain between regimens among men with baseline BMI <25 kg/m^2^.^31^ In contrast, a study from the Women’s Interagency HIV Study (WIHS) reported that BMI <30 kg/m^2^ was a risk factor for greater weight gain among those using INSTI-based regimens.^22^ Similarly, our study found a greater risk of 10%, 20%, or 10 kg weight gain for African American females with baseline BMI <25 kg/m^2^, but not among those with BMI ≥25 kg/m^2^. However, Lake et al found that BMI ≥30 kg/m^2^ at switch was associated with greater weight gain among women.^28^

Unlike female African Americans, mean differences between regimens among Hispanics did not reach statistical significance and contrasting results were observed for subgroups with baseline BMI <25 or ≥25 kg/m^2^. The literature also shows conflicting results regarding risk of weight gain among Hispanics, with reports showing either similar^20,28^ or greater risk^22^ as compared with non-Hispanic whites. As in most of the literature, the present study examined a Hispanic study population that reported a variety of racial backgrounds (approximately 56% white, 40% other/unknown, and 4% African American), and diverse ancestry among Hispanic PLWH has been associated with differing HIV care outcomes.^32^ Indeed, a recent study has shown differences in weight gain within White, Black, or Hispanic cohorts who switch to INSTI-based regimens may be associated with differences in genetic markers related to ancestry; however, the study sample included too few people of Hispanic ethnicity to draw firm conclusions.^33^ Interpretation of our results is also complicated by the inclusion of both females (25%) and males (75%) in the Hispanic study population. As mentioned previously, several studies have reported differences between genders in ARV-related weight gain.^20,28^ Although the sample size of Hispanic PLWH in our study was not sufficient to allow separate analysis of males and females, the impact of sex on weight gain should be explored in future studies. Another factor that may explain the differing results for the female African American and Hispanic cohorts is differences in type and quality of health care received by these two study populations; in the present study, female African Americans were more likely to have Medicare/Medicaid coverage and Hispanics were more likely to have Commercial insurance or Other insurance (which includes none). However, due to the large proportion of missing data for insurance plan type (approximately 50%) in our study population, further research would be required to explore this hypothesis. Lastly, in our study, the Hispanic cohort was younger, had fewer comorbidities, and had lower baseline weight than the African American females, which may also have affected the differences in weight gain.

It is possible that differences in weight gain between regimens may be related to differences in “return to health,” a well-documented phenomenon among newly treated PLWH.^20,34^ However, our cohorts were well-balanced on demographic and clinical variables and differences between regimens related to return-to-health would be expected to be minor. Moreover, only 3%-6% of our study population was underweight at time of ARV initiation, which has been reported to be the strongest predictor for return-to-health weight gain.^35^ In addition, follow-up weight/BMI measures were required to be in the fourth month or later after the index date, thus excluding any transient weight gain that occurs immediately after ARV initiation. Lastly, a WIHS study that excluded women who were ARV naïve or had HIV RNA levels ≥1000 copies/ml also reported greater weight gain among those who switched to INSTI-based regimens, suggesting that differences in weight gain between regimens are not dependent on return to health.^22^ Future studies may shed light on this phenomenon by assessing weight changes in treatment-naïve populations stratified by baseline weight and HIV disease state using currently prescribed regimens.

As previously mentioned, weight gain among PLWH is associated with an increased risk of serious conditions such as diabetes, MI, and stroke.^5,8^ In addition to its direct impacts on health, increased weight gain may have a negative impact on adherence to ARV regimens. For example, a 2009 study using WIHS data showed that self-perception of fat gain in the abdomen was the strongest predictor of nonadherence to ARV regimens, especially among African American females.^36^ This is especially concerning since adherence to ARV regimens has been shown to be lower among non-White PLWH (especially African Americans) as compared with White PLWH,^37–39^ and low adherence is known to be associated with decreased viral suppression and increased risk of drug resistance.^39^ Lastly, regardless of HIV status, excess body weight is associated with higher health care costs,^40^ thus contributing to additional burden for women and minorities.

This real-world study supplements the current literature by focusing on subgroups of PLWH (female African Americans and Hispanics), who are at high risk of weight gain due to the presence of certain comorbidities (hypertension, obesity, and type 2 diabetes) and other health care disparities. The Optum^®^ EHR database comprises data from a geographically diverse group of provider networks in the United States and includes variables (eg, bodyweight, BMI) that are often absent from other real-world data sources. This study measured a variety of clinically meaningful endpoints such as absolute weight/BMI increase, proportion with weight gain ≥10 kg, ≥5%, ≥10%, or ≥20% of total body weight, and assessed outcomes overall and by BMI category. Another strength of this study is the IPTW approach, which was used to control for selection bias and improve cohort comparability. IPTW was performed separately for treatment cohorts with and without TAF and to control for the effect of TAF, which has been independently associated with weight gain.^20,23^ Consistent with the literature, we observed greater weight gain among patients initiating regimens with TAF vs without TAF (for both INSTI- or PI-based regimens), although the number of patients using PI-based regimens with TAF was insufficient to perform a formal comparison. Studies with a larger sample size are needed to assess the impact on weight gain for TAF vs tenofovir isoproxil fumarate in ARV regimens.

This study has some limitations. Prescription records from EHR do not necessarily indicate whether the patient filled a prescription or took the medication. Although steps were taken to ensure patients were newly initiating ARV, the first HIV diagnosis or ARV prescription observed in the database may not correspond to the patient’s first diagnosis, since patients may have had gaps in care or switched from a provider that does not provide data to the Optum^®^ EHR database. In addition, there are various factors that may impact treatment choice and/or risk of weight gain, such as income, education, geographic location, rural/urban location, and behavioral factors, but that are not available in the data and could not be controlled. However, we included in the model key factors that would be expected to impact the choice of HIV treatment, namely, comorbid conditions and concomitant medications. Lastly, due to sample size limitations, composite scores were used to reflect aggregated drug classes, which may have masked differences between individual drugs.

## CONCLUSIONS

In this observational study using EHR from a large, geographically representative US database, INSTI-based regimens were associated with greater absolute weight gain and greater risk of 5% weight gain among treatment-naïve female African Americans vs PI-based regimens. Among Hispanics, risk of 5% weight gain was greater for INSTI-based vs PI-based regimens, but changes in absolute weight gain were not significantly different. This study also demonstrated a high prevalence of obesity and other cardiovascular/metabolic comorbidities among female African Americans and Hispanics, which are likely to have an important health impact in these high-risk populations. Although additional studies with greater sample sizes and longer follow-up time are needed, the present results support recommendations to monitor weight changes among PLWH initiating INSTI-based regimens and may also inform treatment choice for patients who are at risk for ARV-related weight gain.

### Author Contributions

Drs. Y-WC, DA, CDP, and PD were involved in study design, analysis, and interpretation. Y-WC performed the statistical analyses. All authors had full access to all study data and take responsibility for data integrity and accuracy of data analysis. All authors meet ICMJE criteria and all those fulfilling criteria are listed as authors. All authors provided input on the manuscript, made the final decision about where to publish these data, and approved submission to this journal.

### Conflicts of Interest

Drs. Y-WC, DA, CDP, and PD are employees of Janssen Scientific Affairs, LLC. All authors hold stock in Johnson & Johnson, of which Janssen Scientific Affairs is a wholly-owned subsidiary.

## Supplementary Material

Appendices: Code Lists

Online Supplemental Material
