# Supplementary material for: Real-World Assessment of Weight Change in African American Females and Hispanics with HIV-1 After Initiating Integrase Strand-Transfer Inhibitors or Protease Inhibitors
Source: J Health Econ Outcomes Res. 2022 Jan 3;9(1):1–10. doi: 10.36469/001c.30184 (PMC8723886; doi:10.36469/001c.30184)
Supplement: Online Supplemental Material [file jheor_2022_9_1_30184_78356.pdf]

Journal of Health Economics and Outcomes Research

Online Supplementary Material

Real-World Assessment of Weight Change in African American Females and Hispanics with HIV-1 After Initiating Integrase Strand Transfer Inhibitors or Protease Inhibitors. *JHEOR*. 2022;9(1):1-10. doi:10.36469/jheor.2022.30184

Figure S1. Mean Weight and BMI Changes in Female African American Patients

Figure S2. Odds Ratio of Weight Gain for Female African American Patients

Figure S3. Mean Weight and BMI Changes in Hispanic Patients

Figure S4. Odds Ratio of Weight Gain for Hispanic Patients

Table S1. Demographics for Unweighted/Weighted, With TAF Subgroups and P Values (Female African American)

Table S2. Demographics for Unweighted/Weighted, With TAF Subgroups and P Values (Hispanic)

This supplementary material has been provided by the authors to give readers additional information about their work.

This is an open-access article distributed under the terms of the Creative Commons Attribution 4.0 International License (CCBY-4.0). View this license's legal deed at http://creativecommons.org/licenses/by/4.0 and legal code at http://creativecommons.org/licenses/by/4.0/legalcode for more information.

Figure S1. Mean Weight and BMI Changes in Female African American Patients

A. BMI <25

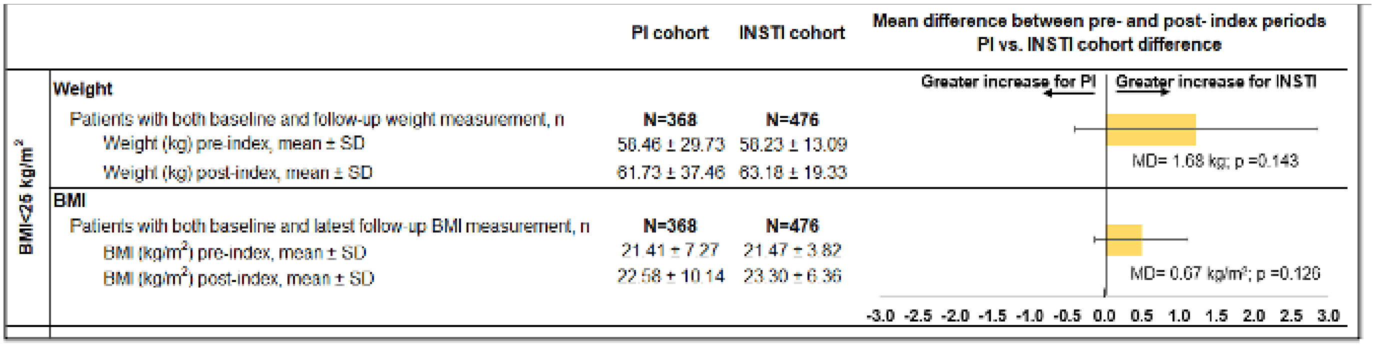

B. BMI ≥25

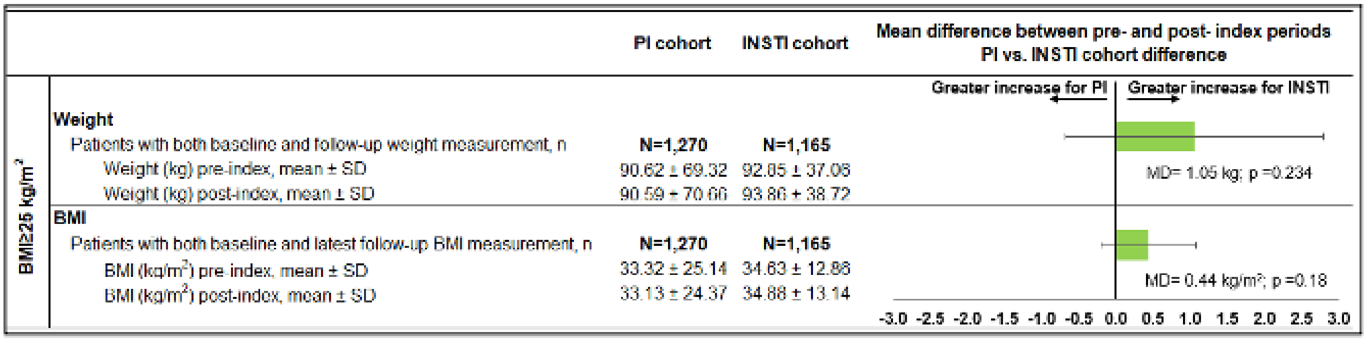

Figure S2. Odds Ratio of Weight Gain for Female African American Patients

A. BMI <25 (PI, n=368; INSTI, n=476)

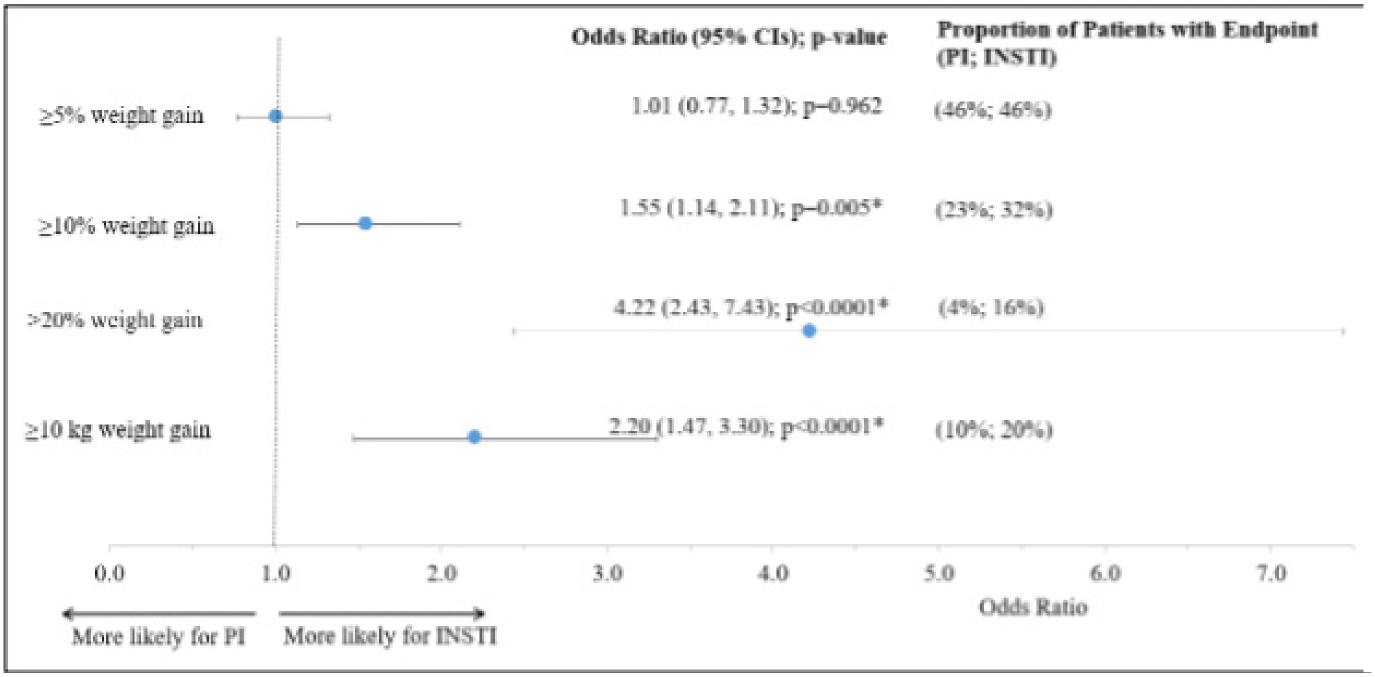

B. BMI ≥25 (PI, n=1270; INSTI, n=1165)

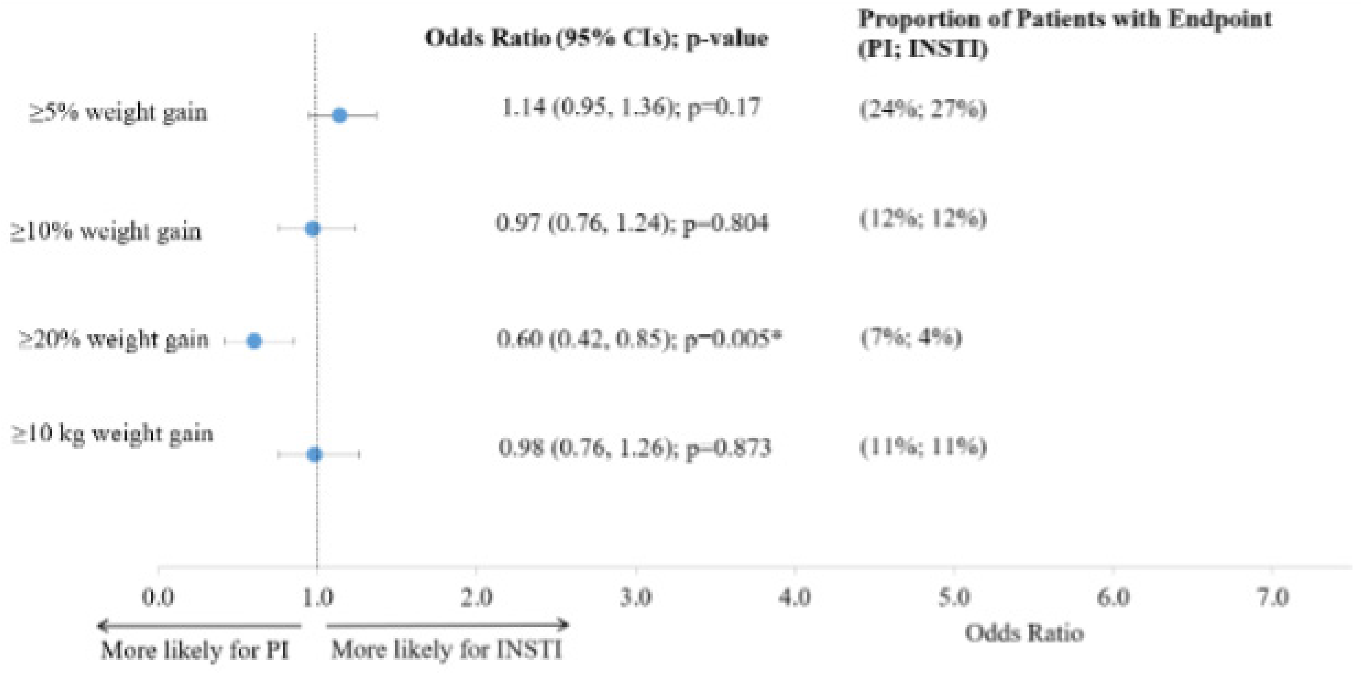

Figure S3. Mean Weight and BMI Changes in Hispanic Patients

A. BMI <25

BMI<25 kg/m²		PI cohort	INSTI cohort	Mean difference between pre- and post- index periods PI vs. INSTI cohort difference
	**Weight**			
	Patients with both baseline and follow-up weight measurement, n	N=633	N=687	
	Weight (kg) pre-index, mean ± SD	61.09 ± 39.98	63.94 ± 14.47	MD= -1.73 kg; p =0.035*
	Weight (kg) post-index, mean ± SD	65.30 ± 50.03	66.42 ± 16.24	
	**BMI**			
	Patients with both baseline and latest follow-up BMI measurement, n	N=633	N=687	
	BMI (kg/m²) pre-index, mean ± SD	21.76 ± 9.18	22.05 ± 3.47	MD= -0.42 kg/m²; p =0.158
	BMI (kg/m²) post-index, mean ± SD	23.13 ± 12.61	23.00 ± 4.47	

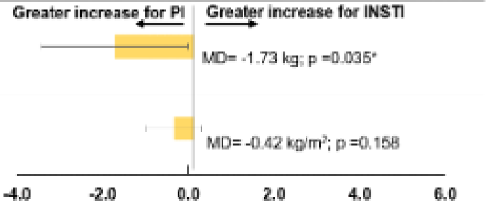

BMI = body-mass index; INSTI = integrase strand transfer inhibitor; MD = mean difference; PI = protease inhibitor; SD = standard deviation.
* Indicates p<0.05.

B. BMI ≥25

BMI≥25 kg/m²		PI cohort	INSTI cohort	Mean difference between pre- and post- index periods PI vs. INSTI cohort difference
	**Weight**			
	Patients with both baseline and follow-up weight measurement, n	N=1,118	N=1,110	
	Weight (kg) pre-index, mean ± SD	85.61 ± 68.88	87.25 ± 25.22	MD= 1.62 kg; p =0.005*
	Weight (kg) post-index, mean ± SD	85.94 ± 74.63	89.21 ± 28.06	
	**BMI**			
	Patients with both baseline and latest follow-up BMI measurement, n	N=1,118	N=1,110	
	BMI (kg/m²) pre-index, mean ± SD	30.33 ± 19.68	30.70 ± 8.21	MD= 0.45 kg/m²; p =0.043*
	BMI (kg/m²) post-index, mean ± SD	30.41 ± 22.68	31.24 ± 9.28	

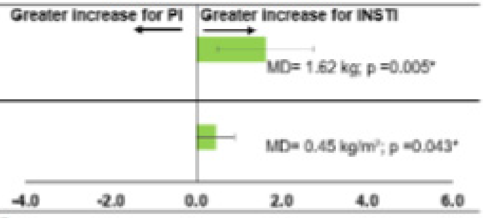

BMI = body-mass index; INSTI = integrase strand transfer inhibitor; MD = mean difference; PI = protease inhibitor; SD = standard deviation.
* Indicates p<0.05.

Figure S4. Odds Ratio of Weight Gain for Hispanic Patients

A. BMI <25 (PI, n=633; INSTI, n=687)

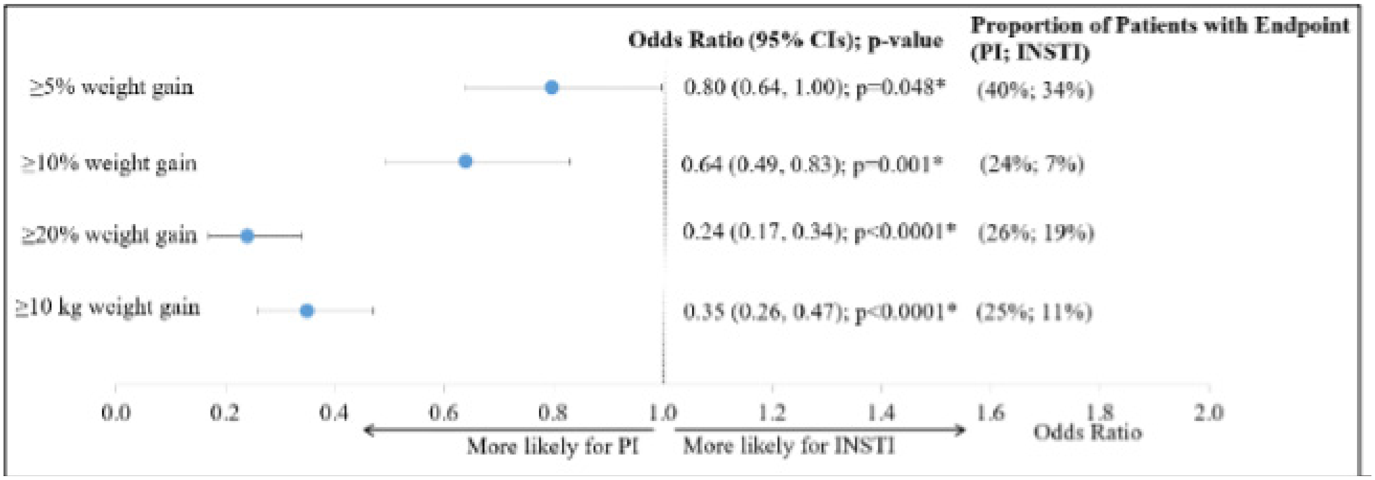

B. BMI ≥25 (PI, n=1118; INSTI, n=1110)

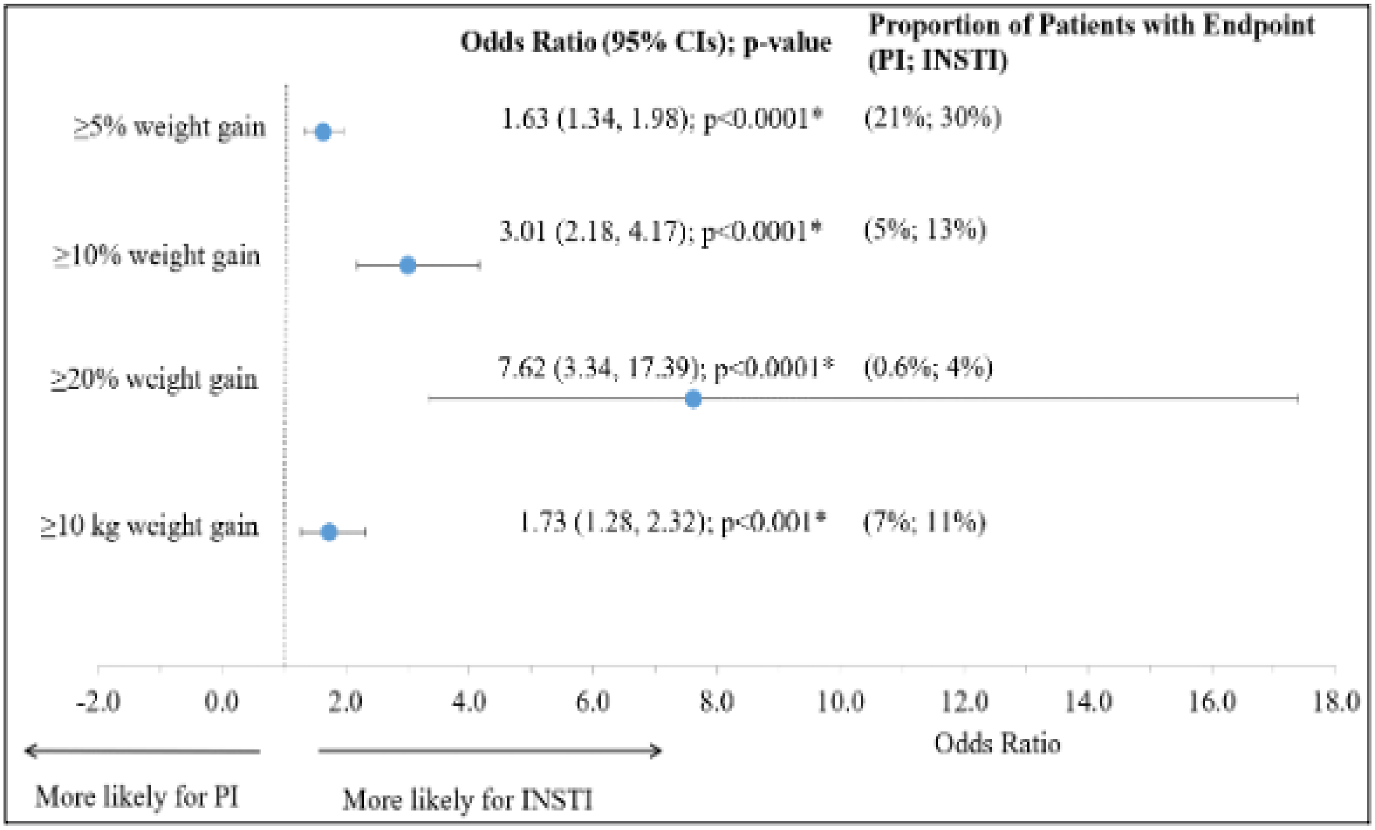

Abbreviations: BMI, body-mass index; CIs, confidence intervals; INSTI, integrase strand transfer inhibitor; PI, protease inhibitor.

*Indicates p<0.05.

Table S1. Baseline Demographic and Clinical Characteristics, Unweighted and Weighted, Female African Americans

Parameter[a]	Unweighted					Weighted				
	Drug Class				*P* Value	Drug Class				*P* Value
	PI w/o TAF (n=130)	PI + TAF (n=34)	INSTI w/o TAF (n=472)	INSTI + TAF (n=216)		PI w/o TAF (n=849)	PI + TAF (n=854)	INSTI w/o TAF (n=852)	INSTI + TAF (n=851)	
Age, mean (SD)	48.3 (9.3)	47.2 (12.0)	46.4 (10.9)	47.1 (11.7)	0.368	47.5 (9.1)	46.9 (11.4)	46.9 (10.8)	46.9 (11.7)	0.935
Baseline weight, mean (SD)	80.5 (22.7)	83.4 (21.1)	83.0 (26.2)	83.2 (25.1)	0.772	82.9 (23.8)	83.6 (21.4)	82.6 (25.9)	82.9 (24.9)	0.980
Baseline BMI, mean (SD)	29.8 (8.4)	30.9 (7.3)	31.2 (9.7)	30.7 (8.9)	0.537	30.7 (9.2)	30.6 (7.3)	31.1 (9.6)	30.6 (8.8)	0.937
QCI score, mean (SD)	2.4 (2.3)	2.9 (2.6)	3.3 (2.3)	3.3 (2.2)	0.001	3.2 (2.4)	3.2 (2.6)	3.2 (2.3)	3.2 (2.2)	0.996
CVD conditions, mean (SD)[b]	0.6 (0.9)	0.8 (1.2)	0.8 (1.0)	0.8 (1.0)	0.451	0.8 (1.0)	0.8 (1.1)	0.8 (1.0)	0.8 (1.0)	0.965
Individual conditions, n (%)										
Type 2 diabetes mellitus	14 (10.8)	7 (20.6)	76 (16.1)	36 (16.7)	0.359	131 (15.4)	146 (17.1)	133 (15.6)	130 (15.3)	<0.0001
Hypertension	41 (31.5)	10 (29.4)	175 (37.1)	80 (37.0)	0.552	314 (37.0)	208 (24.3)	309 (36.2)	311 (36.5)	<0.0001
Hyperlipidemia	18 (13.9)	5 (14.7)	66 (14.0)	33 (15.3)	0.972	144 (17.0)	103 (12.0)	116 (13.6)	128 (15.0)	<0.0001
AIDS	66 (50.8)	18 (52.9)	321 (68.0)	152 (70.4)	0.000	549 (64.7)	525 (61.5)	555 (65.1)	573 (67.3)	0.810
Baseline drugs, mean (SD)[c]	0.5 (1.0)	0.5 (0.9)	0.7 (1.2)	0.8 (1.3)	0.181	0.6 (1.1)	0.6 (1.0)	0.7 (1.2)	0.7 (1.2)	0.986

Abbreviations**:** BMI, body mass index; INSTI, integrase inhibitors; PI, protease inhibitors; QCI, Quan-Charlson Comorbidity Index; TAF, tenofovir alafenamide.

[a] Propensity score was calculated based on age, baseline weight, baseline QCI score, the number of CVD risk factors (including pre-diabetes, type 2 diabetes mellitus, myocardial infarction, congestive heart failure, hypertension and hyperlipidemia) at baseline, and the number of drugs used at baseline.

[b]CVD conditions included pre-diabetes, type 2 diabetes mellitus, myocardial infarction, congestive heart failure, hypertension, and hyperlipidemia.

[c]Included the following potentially weight modifying drugs: diabetes therapies, psychiatric/neurologic therapies, hormone therapy/contraception, stimulants/appetite suppressants, and anti-hypertensives.

Table S2. Baseline Demographic and Clinical Characteristics, Unweighted and Weighted, Hispanics

Parameter[a]	Unweighted					Weighted				
	Drug Class				*P* Value	Drug Class				*P* Value
	PI w/o TAF (n=119)	PI + TAF (n=30)	INSTI w/o TAF (n=480)	INSTI + TAF (n=303)		PI w/o TAF (n=919)	PI + TAF (n=927)	INSTI w/o TAF (n=932)	INSTI + TAF (n=933)	
**Age, mean (SD)**	48.0 (10.4)	41.8 (11.2)	42.0 (13.0)	42.9 (13.2)	<0.0001	44.1 (10.8)	42.6 (10.9)	43.1 (13.2)	43.0 (13.3)	0.553
**Female, n (%)**	32 (26.9)	3 (10.0)	119 (24.8)	75 (24.8)	0.272	244 (26.5)	231 (24.9)	231 (24.8)	231 (24.7)	0.002
**Race, n (%)**										
African American	7 (5.9)	0 (0.0)	26 (5.4)	16 (5.3)	0.736	51 (5.5)	0 (0.0)	51 (5.4)	51 (5.5)	<0.0001
Asian	0 (0.0)	0 (0.0)	2 (0.4)	1 (0.3)	0.893	0 (0.0)	0 (0.0)	4 (0.4)	3 (0.3)	<0.0001
Caucasian	55 (46.2)	24 (80.0)	248 (51.7)	156 (51.5)	0.010	444 (48.3)	682 (73.6)	478 (51.2)	481 (51.6)	<0.0001
Other/Unknown	57 (47.9)	6 (20.0)	204 (42.5)	130 (42.9)	0.048	425 (46.2)	245 (26.4)	400 (43.0)	398 (42.7)	<0.0001
**Baseline weight, mean (SD)**	79.8 (21.7)	76.1 (17.8)	78.4 (18.6)	78.4 (17.8)	0.777	77.7 (20.6)	76.7 (19.7)	78.4 (18.5)	78.7 (17.8)	0.678
**Baseline BMI, mean (SD)**	27.5 (6.9)	27.0 (5.2)	27.3 (5.9)	27.3 (6.2)	0.982	26.9 (6.5)	27.5 (5.8)	27.4 (5.9)	27.4 (6.2)	0.705
**QCI score, mean (SD)**	3.4 (2.1)	2.7 (1.9)	3.0 (2.1)	3.2 (2.2)	0.166	3.0 (2.2)	3.2 (2.0)	3.1 (2.1)	3.1 (2.2)	0.852
**CVD conditions, mean (SD)[b]**	0.6 (0.9)	0.4 (0.9)	0.5 (0.9)	0.5 (0.9)	0.646	0.5 (0.8)	0.6 (1.0)	0.5 (0.9)	0.5 (0.9)	0.162
**Individual conditions, n (%)**										
Type 2 diabetes mellitus	15 (12.6)	2 (6.7)	50 (10.4)	44 (14.5)	0.305	104 (11.3)	135 (14.5)	106 (11.4)	126 (13.5)	0.001
Hypertension	26 (21.9)	6 (20.0)	95 (19.8)	54 (17.8)	0.786	139 (15.1)	177 (19.2)	176 (18.9)	134 (14.4)	0.030
Hyperlipidemia	27 (22.7)	3 (10.0)	74 (15.4)	50 (16.5)	0.2008	158 (17.2)	179 (19.3)	158 (16.9)	149 (16.0)	0.001
AIDS	89 (74.8)	18 (60.0)	325 (67.7)	211 (69.6)	0.3296	602 (65.5)	620 (66.7)	646 (69.3)	637 (68.3)	0.000
**Baseline drugs, mean (SD)[c]**	0.4 (0.7)	0.3 (0.6)	0.4 (0.9)	0.5 (0.9)	0.452	0.4 (0.7)	0.4 (0.7)	0.4 (0.9)	0.4 (0.8)	0.863

Abbreviations: BMI, body mass index; INSTI, integrase inhibitors; PI, protease inhibitor; QCI, Quan-Charlson Comorbidity Index; TAF, tenofovir alafenamide.

[a] Propensity score was calculated based on age, reported gender, baseline weight, baseline QCI score and the number of drugs used at baseline.
[b] CVD conditions included pre-diabetes, type 2 diabetes mellitus, myocardial infarction, congestive heart failure, hypertension, and hyperlipidemia.
[c] Included the following potentially weight modifying drugs: diabetes therapies, psychiatric/neurologic therapies, hormone therapy/contraception, stimulants/appetite suppressants, and anti-hypertensives.
